# Regulation of Light Spectra on Cell Division of the Unicellular Green Alga *Haematococcus pluvialis*: Insights from Physiological and Lipidomic Analysis

**DOI:** 10.3390/cells11121956

**Published:** 2022-06-17

**Authors:** Kuo Zhao, Yanhua Li, Hailong Yan, Qiang Hu, Danxiang Han

**Affiliations:** 1 Center for Microalgal Biofuels and Biotechnology, Institute of Hydrobiology, Chinese Academy of Sciences, Wuhan 430072, China; kuozhao@foxmail.com (K.Z.); yanhuali@ihb.ac.cn (Y.L.); 2 College of Advanced Agricultural Sciences, University of Chinese Academy of Sciences, Beijing 100086, China; 3 Institute for Advanced Study, Shenzhen University, Shenzhen 518060, China; hailong-yan@hotmail.com (H.Y.); huqiang@szu.edu.cn (Q.H.)

**Keywords:** *Haematococcus pluvialis*, cell division, lipidome, light-emitting diodes, light spectra, green stage

## Abstract

Commercial scale production of natural astaxanthin is currently conducted through cultivation of the green alga *Haematococcus pluvialis*. This study comprehensively investigated the impact of seven different light spectra on the growth, morphology and photosynthesis of *H. pluvialis* vegetative cells. Further, the lipidomes of vegetative *H. pluvialis* grown under various light spectra were qualitatively and quantitatively analyzed using liquid chromatography/mass spectrometry (LC/MS). The results showed the existence of blue light—alone or with red light—promoted cell division, while pure red light or white light enabled increased cell sizes, cellular pigment, starch and lipid contents, and biomass production. Although the photosynthetic performance of *H. pluvialis* measured as chlorophyll *a* fluorescence was not significantly affected by light spectra, the lipid profiles, particularly chloroplast membrane lipids, showed remarkable changes with light spectra. The contents of most lipid species in the blue/red light 1/2 group, which showed the fastest cell division, remained at a moderate level compared with those under other light spectra, indicating the fastest dividing cells were featured by a fine-tuned lipid profile. From biotechnical perspective, this comprehensive study can provide insights into the development of appropriate light regimes to promote the cell density or biomass of *H. pluvialis* mass culture.

## 1. Introduction

Astaxanthin is widely used in the animal feed, food, pharmaceuticals and cosmetics sectors due to its red color and strong antioxidant properties [1,2]. The astaxanthin used in the latter three sectors is currently dominated by natural ones [3,4], whereas that in the former sector is dominated by chemically synthesized ones [5,6]. To date, the large-scale production of natural astaxanthin is achieved through the cultivation of one green alga, *Haematococcus pluvialis* [5,7]. *H. pluvialis* is a freshwater unicellular green microalga characterized by a complex life history [8]. Under favorable culture conditions, green motile cells with two flagella are dominant. As the culture conditions become unfavorable (e.g., high light, nitrogen deficiency, etc.), *H. pluvialis* cells lose their flagella, increase cell size, form a rigid cell wall and accumulate red astaxanthin, eventually forming nonmotile red aplanospores. With the relief from the environmental stresses, the nonmotile aplanospores are able to divide and germinate.

Based on the life history of *H. pluvialis*, a two-stage cultivation strategy has been developed and is widely used in the large-scale cultivation of *H. pluvialis* to produce astaxanthin. The two-stage cultivation includes a green stage and a red stage. In the green stage, favorable culture conditions are provided so that *H. pluvialis* cells maintain a green motile status and divide rapidly. In the red stage, stress conditions such as high light, nitrogen deficiency, high salinity, etc., are applied to induce cells to accumulate astaxanthin, which eventually turn into red nonmotile aplanospores.

The vegetative green cells of *H. pluvialis* suffer from a low growth rate, especially compared with many other production microalgal strains such as *Chlorella vulgaris* and *Dunaliella salina* [9]. Light source has long been considered the most important abiotic factor that affects the growth of microalgal cells under photoautotrophic mode. Compared with traditional artificial light sources, including incandescent and gas-discharge lamps (e.g., fluorescent lamps), the new-generation light source light-emitting diodes (LEDs) can generate artificial light with a selected peak wavelength, and the light intensity can be easily adjusted by changing current [10]. In other words, LEDs allow for better control over light quality and intensity than those conventional artificial light sources. LEDs also have advantages in terms of luminous efficiency, power requirements, life span, heat generation, dimming, directionality, robustness and safety over other artificial light-sources [10].

With the development of LED lighting technology, more and more studies on the impact of light quality on growth, morphology, physiology and metabolism of microalgae are available. Compared with blue light, red light seems to be more conducive to the growth of *H. pluvialis* [11,12,13,14]. Nonetheless, red light does not necessarily have advantages over mixed light spectra in promoting vegetative growth of *H. pluvialis* [15,16,17]. Overall, no consensus has currently been reached on the optimal light wavelength for the cultivation of *H. pluvialis* vegetative cells. In addition, morphological changes have been observed in microalgae grown under various wavelengths of light. A number of green microalgae, including *Chlorella* sp. [18,19,20], *Chlamydomonas reinhardtii* [21,22] and *Dunaliella salina* [23], coincidentally exhibit larger cell sizes under blue light than under red light. Further, the impacts of light spectra on the biochemical composition of microalgal cells are of particular interest. Lipids are a group of macromolecules that constitute plastid membranes and extraplastidic membranes in microalgae [24]. Thus, they are assumed to be susceptible to changing light regimes, including light spectra. Analysis of lipidomes will possibly provide clues to elucidate the impact of different light spectra on the growth, morphological and physiological variations of *H. pluvialis* cells under environmental stimuli.

Therefore, this study aimed to comprehensively investigate the impact of seven different light spectra on the growth and morphology of *H. pluvialis* green cells. Then, chlorophyll a fluorescence was measured to evaluate the photosynthetic performance of the cells grown under different light spectra. Further, liquid chromatography coupled with mass spectrometry (LC/MS), a widely used method for lipidomic analysis, was performed to identify and quantify several major membrane glycerolipids (particularly for chloroplast membrane lipids), including monogalactosyldiacylglycerol (MGDG), digalactosyldiacylglycerol (DGDG), sulfoquinovosyl-diacylglycerol (SQDG), phosphatidylglycerol (PG), phosphatidylethanolamine (PE), phosphatidylcholine (PC), phosphoinositides (PI) and diacylglyceryltrimethylhomo-Ser (DGTS), in *H. pluvialis* green cells grown under different light spectra in a time-course manner, covering both exponential and stationary growth phases. It was found that the division, morphology, photosynthesis and lipid profile of the *H. pluvialis* green cells were significantly influenced by light spectra. This study provided novel insights into the regulatory effects of light spectra on *H. pluvialis* cells at multiple levels, which could facilitate developing appropriate light regimes to promote the efficiency of cultivation for *H. pluvialis*.

## 2. Materials and Methods

### 2.1. Microalgal Strain and Cultivation Conditions

*H. pluvialis* strain CMBB-361 was provided by Center for Microalgal Biotechnology and Biofuels (Institute of Hydrobiology, Chinese Academy of Sciences, Wuhan, China). Seed culture was maintained in the BG11 growth medium in bubble column reactors with an inner diameter of 4.5 cm under aeration (CO_2_/air, *v*/*v* 1%). Temperature was maintained at 22 ± 1 °C. White LEDs (blue LED+ yellow fluorescent material) were used as light source. Continuous light was provided with intensity set at 40 μmol photons m^−2^ s^−^^1^.

Algal cells that were cultivated for about 4 days and in exponential phase were used for inoculation. The inoculation density was 5 × 10^4^ cells/mL, and the initial culture volume was 700 mL. The impact of seven different light spectra (including a narrow band of blue light with wavelength centered around 450 nm, blue/red light 2/1, blue/red light 1/1, blue/red light 1/2, a narrow band of red light with wavelength centered around 660 nm, a narrow band of infrared with wavelength centered around 730 nm and white LED light) on the vegetative growth of *H. pluvialis* were then investigated. For detailed wavelength information, see Figure S1 in the Supplementary Materials. The LED panels used were provided by Taiwan HiPoint Corporation (Model Z3RGB, Taiwan, China). During cultivation, light intensity was set to 40 μmol m^−2^ s^−1^ (an average of light intensities at 10 measuring points on the illuminated surface of the bubble column reactor) and continuous illumination was provided. The temperature was maintained at 22 ± 1 °C. For aeration, a mixture of CO_2_ and air (1%, *v*/*v*) was fed into the culture medium from a tube extending to the bottom of each reactor. During cultivation, the columns were covered with a piece of black cloth to avoid the influence of ambient light.

Three biological replicates were set for each light spectrum. Therefore, unless otherwise stated, the results were presented as mean ± standard deviation (SD) of the measurements of the three biological replicates. Statistical significance was examined by analysis of variance (ANOVA) with Tukey’s multiple comparison tests on GraphPad Prism (v.7.0 for windows, GraphPad Software, Inc., San Diego, CA, USA). A value of *p* < 0.05 was considered statistically significant.

### 2.2. Determination of Cell Number and Dry Cell Weight

During cultivation, samples were taken regularly every day. Cell concentration or number (cells/mL) was counted with a hemocytometer (Paul Marienfeld GmbH & Co., KG, Lauda-Königshofen, Germany) under a microscope (CX31, OLYMPUS Corporation, Tokyo, Japan). For determination of dry cell weight (g/L), a fixed volume (V) of cell cultures was filtered onto a pre-weighed (W_1_) 1.2-μm glass microfiber filter (Whatman International Ltd., Maidstone, UK), which was then placed in an oven set at 105 °C overnight. After being cooled in a dryer, the filter was weighed (W_2_). Then, the dry cell weight of microalgae was calculated as (W_2_ − W_1_)/V.

### 2.3. Analysis of Cell Morphology and Inclusions

Flow cytometric analysis: 1 mL of microalgal sample was collected and passed through a 45 μm filter. The filtered sample was then loaded onto flow cytometer (Cytomics FC500, Beckman Coulter, Inc., Brea, CA, USA) for analysis. For each sample, a total of 20,000 events were analyzed.

Microscopic observation and measurement: Cell morphology was observed and captured with a microscope (BX53, coupled with a DP70 CCD camera, OLYMPUS Corporation, Tokyo, Japan). The diameters of microalgal cells were manually measured using the measuring ruler of the image capture software cellSens Standard included with the microscope and a total of 200 randomly selected cells were measured for each sample. Here, the diameter was referred to as the axis that is perpendicular to the other axis that passes flagellum base.

Photosynthetic pigment analysis: The determination of photosynthetic pigment contents in microalgae was in accordance with [25] with a spectrophotometer (DR6000, HACH Company, Loveland, CO, USA).

Starch content analysis: The starch contents were determined using a Total Starch Assay Kit purchased from Beijing Solarbio Science & Technology Co., Ltd. (Beijing, China) in accordance with the manufacturer’s instructions.

### 2.4. Measurement of Chlorophyll a Fluorescence Parameters

The chlorophyll a fluorescence parameters of the photosystem II of *H. pluvialis* green cells were determined by a dual-channel PAM chlorophyll fluorometer (Dual-PAM 100, Walz Company, Effeltrich, Germany). After adaptation of the microalgal cells in dark for 15 min, the slow kinetics were determined, and chlorophyll a fluorescence parameters such as Fv/Fm (intrinsic or maximum efficiency of photosystem II), Y(II) (efficiency of photosystem II photochemistry) and ETR(II) (linear electron transport rate) of the microalgal cells were obtained from the slow kinetics. Then, the light-response curve of microalgal cells was measured to obtain the trends of Y(II), ETR(II) and NPQ (nonphotochemical quenching) with light intensity.

### 2.5. Lipidome Analysis

Extraction of total lipids was in accordance with previous research [26], with slight modifications. Briefly, 15 mg of lyophilized microalgal powder was weighed into a labeled 12 mL glass tube. Then, 6 mL of extraction solution (methanol:chloroform: 88% formic acid 20:10:1, *v*/*v*) was added to the labeled glass tube (denoted as extraction tube), which was later vortex-mixed for 1 h until the microalgal powder turned white. Subsequently, 3 mL of 0.2 M H_3_PO_4_ + 1 M KCl was added to the extraction tube, which was then vortexed for 30 s to remove protein. After centrifugation at 1000× *g* for 5 min at 25 °C, the organic phase (lower phase) was transferred carefully from the extraction tube to a lipid collection vial. The sample in the collection vial was blow-dried in nitrogen until glistering. The extracted lipids were stored at −80 °C until analysis. Note that the methanol and chloroform used were of high-performance liquid chromatography (HPLC) grade. Formic acid, H_3_PO_4_ and KCl were of analytical grade. The water used was ultrapure deionized distilled water.

Determination of total lipid content: One milliliter of chloroform/methanol (1/1, *v*/*v*, LC-MS grade) was added to the lipid collection vial containing the blow-dried sample. After filtration through organic phase syringe filter (13 mm, 0.22 μm), 500 μL of the sample was taken and put into a pre-dried and weighed 2 mL brown sample vial (W_1_). (Note that the remaining filtered sample would be used for subsequent lipidome analysis) The sample was blow-dried in nitrogen and then the vial containing the blow-dried sample was put in a vacuum freeze-dryer overnight. Subsequently, the vial was taken out and weighed (W_2_). The content of total lipid (mg/mg) per unit dry weight of microalgae was calculated as (W_2_ − W_1_)/7.5. Two biological repeats were used for the determination of total lipid contents.

LC-MS data acquisition: After mixing with corresponding internal standards (for ESI+ ionization mode, MGDG 18:0/8:0 and DGDG 18:0/18:0 purchased from Matreya Company of USA as well as DGTS-d9 16:0/16:0 purchased from Avanti Polar Lipids Company of USA were used; for ESI-ionization mode, PG 17:0/20:4, PE 17:0/14:1 and PI 17:0/20:4 purchased from Avanti Polar Lipids Company were used), lipid samples were analyzed using an ACQUITY ultra-high liquid chromatography (UPLC) system (Waters Corporation, Milford, MA, USA) coupled with triple quadrupole mass spectrometer (Xevo TQ-S, Waters Corporation, Milford, MA, USA) with an electrospray ionization (ESI) source. Lipid components were separated on an ACQUITY UPLC BEH C18 Column (2.1 mm × 50 mm, 1.7 μm particle size, Waters Corporation, Milford, MA, USA) in the UPLC system. The column temperatures were 30 and 35 °C under ESI+ and ESI—ionization modes, respectively. Under the positive ionization mode, the mobile phase A was made of methanol/acetonitrile/water (19/19/2) with 1% (*v*/*v*) ammonium acetate and 0.1% (*v*/*v*) formic acid, and mobile phase B was made of isopropanol with 1% (*v*/*v*) ammonium acetate and 0.1% (*v*/*v*) formic acid. The gradient elution program was set as follows: 0, 10% B; 1 min, 10% B; 6 min, 25% B; 10 min, 60% B; 10.1 min, 10% B; 13 min, 10% B. DGTS and PC were analyzed as [M + H]+, whereas MGDG and DGDG were detected as [M + NH_4_]^+^. Under the negative ionization mode, the mobile phase A was composed of 85% methanol solution with 1% ammonium acetate, and the mobile phase B was composed of isopropanol with 1% ammonium acetate. The gradient elution program was set as follows: 0 min, 20% B; 1 min, 20% B; 8 min, 40% B; 9 min, 80% B; 11 min, 20% B; 14 min, 20% B. SQDG, PG, PI and PE were analyzed as [M − H]^−^. Flow rate was set to 0.2 mL/min. Injection volume was 1 μL, and the temperature of the sample chamber was set to 4 °C. Note that methanol, acetonitrile, isopropanol, formic acid and ammonium acetate were of LC-MS grade. The water used was ultrapure deionized distilled water.

Lipid identification, quantification and statistical analysis: Precursor ion and neutral loss scanning modes were employed to identify lipid species for a given class according to previously reported methods [27,28,29]. Product ion mode was used to resolve the acyl groups of each lipid species. For quantification purposes, calibration standards of each lipid class were titrated relative to a constant amount of internal standards. The peak intensity ratios of calibration standards relative to internal standards were plotted against their concentration ratios to establish the standard curve. Data were processed using Micro-mass MassLynx v 4.1 (Waters Corporation, Milford, MA, USA) and further processed with Microsoft Excel 2016 (Microsoft, Redmond, WA, USA). Difference between groups was examined by analysis of variance (ANOVA) with Tukey’s multiple comparison tests on GraphPad Prism v.7.0 (GraphPad Software, Inc., San Diego, CA, USA). Heat map was plotted with Origin 2022 (OriginLab, Northampton, MA, USA).

## 3. Results

### 3.1. Effects of Light Spectra on the Growth of H. pluvialis Green Cells

**Figure 1:**
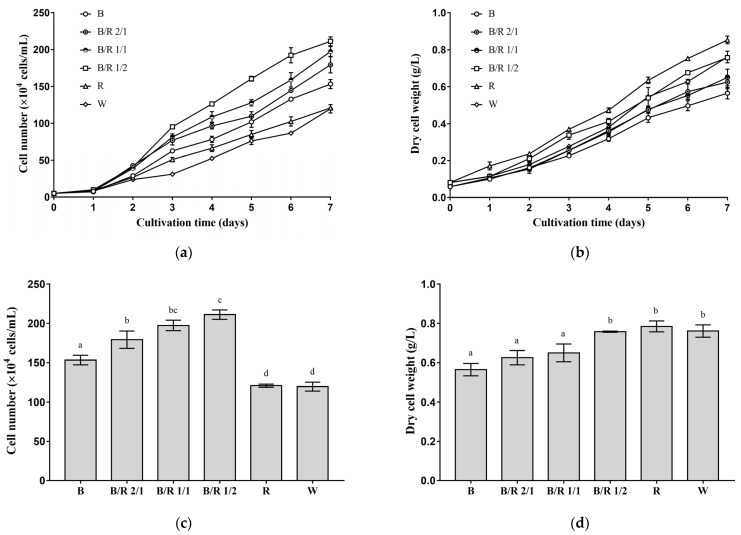
Effects of light spectra on the growth of *H. pluvialis* green cells. (**a**) Growth curves based on cell number; (**b**) growth curves based on dry cell weight; (**c**) cell number obtained at the end of cultivation; (**d**) dry cell weight obtained at the end of cultivation. In (**c**,**d**), the same lowercase letter means insignificant difference, while different lowercase letters mean significant difference. In (**a**–**d**), B—blue light; B/R—blue light/red light; R—red light; W—white light. Results were presented as mean ± SD of the measurements of three biological replicates.

The effects of seven different light spectra, including blue light, blue/red light 2/1, blue/red light 1/1, blue/red light 1/2, red light, white light and infrared, on the vegetative growth of *H. pluvialis* cells were investigated. Under all light spectra (except infrared), the cell number and dry cell weight of *H. pluvialis* green cells increased with cultivation time (Figure 1a,b). Under infrared light, *H. pluvialis* green cells hardly grew, and the cell number and dry cell weight increased little with the extension of cultivation time (data not shown).

Among the several light spectra investigated in this study, blue/red light 1/2 was the most favorable for the division of *H. pluvialis* green cells, followed by blue/red light 1/1, blue/red light 2/1, blue light, red light and white light (Figure 1a). As the blue/red ratio increased, the cell number of *H. pluvialis* cultivated for the same time period first increased and then decreased, with the highest cell number obtained at a blue/red ratio of 1/2 (Figure 1a,c). On the seventh day of cultivation (Figure 1c), the cell number of *H. pluvialis* cultivated under the optimal blue/red 1/2 light condition reached 211 ± 5 × 10^4^ cells/mL, which was about 70% higher than those cultivated under white light (127 ± 3 × 10 ^4^ cells/mL) and red light (121 ± 2 × 10^4^ cells/mL) (*p* < 0.0001) and about 40% higher than that cultivated under blue light (153 ± 5 × 10^4^ cells/mL) (*p* < 0.0001). Additionally, note that the cell number of *H. pluvialis* cultivated under blue light was about 30% higher than that cultivated under red light (*p* < 0.001).

From another perspective, however, among the several light spectra investigated in this study, red light was most favorable for the dry weight accumulation of *H. pluvialis* green cells, followed by blue/red light 1/2, blue/red light 1/1, blue/red light 2/1 and blue light (Figure 1b). Notably, compared with other light spectra, white light enabled only a low level of dry weight accumulation of *H. pluvialis* in the early stage of cultivation. In the late stage of cultivation, however, the dry cell weight of *H. pluvialis* cultivated under white light increased to be higher than those cultivated under most of other spectra. A general trend was identified: as the blue/red ratio decreased, the dry cell weight of *H. pluvialis* cultivated for the same time period increased, with the highest dry cell weight obtained at pure red light (Figure 1b,d). On the seventh day of cultivation (Figure 1d), the dry cell weight of *H. pluvialis* cultivated under the optimal red light reached 0.785 ± 0.023 g/L, a little higher than those obtained under blue/red light 1/2 (0.757 ± 0.002 g/L) and white light (0.761 ± 0.026 g/L) (*p* > 0.05) but about 40% higher than that obtained under blue light (0.565 ± 0.025 g/L, *p* < 0.0001).

In summation, *H. pluvialis* green cells tended to rapidly divide under mixed blue/red light and blue light, while they massively accumulated dry weight under pure red light and white light, suggesting the existence of blue light, alone or with red light, can promote cell division.

### 3.2. Effects of Light Spectra on the Morphology and Inclusions of H. pluvialis Green Cells

**Figure 2:**
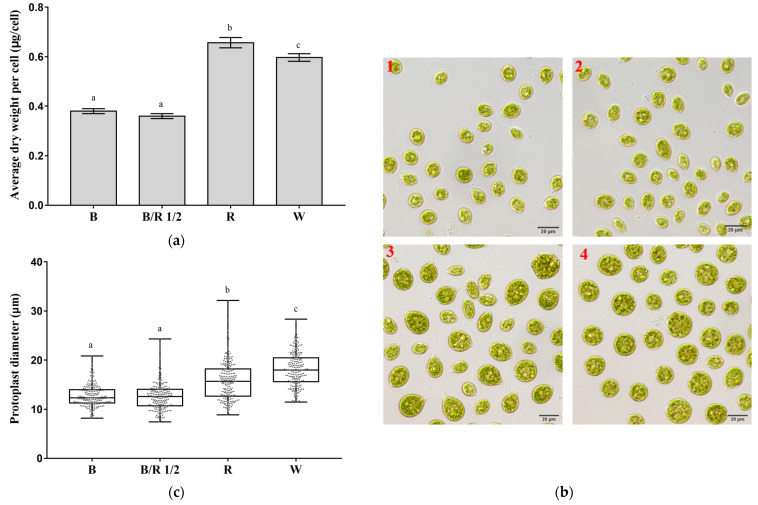
Effects of light spectra on the sizes of *H. pluvialis* green cells. (**a**) Average dry weight per cell at the end of cultivation; (**b**) micrographs of the cells at the end of cultivation with scale bar of 20 μm: (**1**) Blue light; (**2**) blue/red light 1/2; (**3**) red light; (**4**) white light. (**c**) Distribution of protoplast diameters at the end of cultivation (a total of 200 randomly selected cells were measured manually for each light condition). In (**a**,**c**), the same lowercase letter means insignificant difference, while different lowercase letters mean significant difference. In (**a**,**c**), B—Blue light; B/R 1/2—blue/red light 1/2; R—red light; W—white light.

Four typical light spectra (blue light, blue/red light 1/2, red light and white light) were selected to investigate how they influenced the morphology and inclusions of *H. pluvialis* green cells. As shown in Figure 2a, the average dry weights per green cell of *H. pluvialis* grown under red and white light were significantly higher than those grown under blue light and blue/red light 1/2 (*p* < 0.0001). This suggested that the *H. pluvialis* green cells grown under red and white light possibly possessed larger sizes than those grown under blue light and blue/red light 1/2. Microscopic observations showed that the cell sizes of *H. pluvialis* (to be more accurate, the sizes of protoplasts without extracellular matrix) grown under red and white light were indeed larger than those grown under blue light and blue/red light 1/2 (Figure 2b). The micrographs also suggested that *H. pluvialis* green cells grown under red light showed a scattered cell-size distribution, whereas those grown under other lights seemed to be more uniform in cell-size distribution. In addition to cell sizes, it was worth noting that *H. pluvialis* green cells grown under white light tended to present spherical shapes, whereas those grown under other lights (especially red light) were more pear-shaped (Figure 2b). Further measurements revealed that the *H. pluvialis* green cells grown under red light (15.8 ± 3.8 μm) and white light (18.2 ± 3.4 μm) had significantly larger protoplasts than those grown under blue light (12.7 ± 2.1 μm) and blue/red light 1/2 (12.7 ± 2.8 μm) (*p* < 0.0001, Figure 2c). These results could help explain why the cell numbers of *H. pluvialis* grown under red and white light were low, but the dry cell weights were quite high.

**Figure 3:**
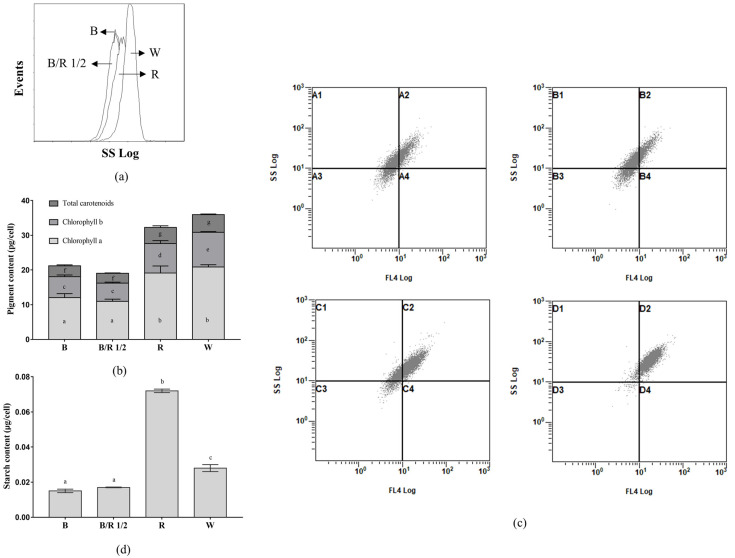
Effects of light spectra on the intracellular granularity and photosynthetic pigment contents of *H. pluvialis* green cells. (**a**) Distribution of SSC signals of cells at the end of cultivation; (**b**) photosynthetic pigment contents in cells at the end of cultivation. For each pigment class, the same lowercase letter means insignificant difference, while different lowercase letters mean significant difference; (**c**) 2D scatter plot of FL4 signals (chlorophyll autofluorescence) vs. SSC signals (intracellular granularity), upper left—blue light; upper right—blue/red light 1/2; lower left—red light; lower right—white light. (**d**) Starch contents in cells at the end of cultivation. The same lowercase letter means insignificant difference, while different lowercase letters mean significant difference. In (**a**,**b**,**d**), B—Blue light; B/R 1/2—Blue/Red light 1/2; R—Red light; W—White light. In (**a**,**c**), a total of 20,000 cells were analyzed by flowcytometry for each light condition.

Further, *H. pluvialis* green cells cultivated under red and white light had higher intracellular granularity than those cultivated under blue light and blue/red light 1/2 (Figure 3a). The contents of chlorophyll a, chlorophyll b and total carotenoids of *H. pluvialis* green cells cultivated under red and white light were also higher than those cultivated under blue light and blue/red light 1/2 (*p* < 0.005, Figure 3b). This suggested that when the intracellular photosynthetic pigment content of *H. pluvialis* was high, the intracellular granularity would be correspondingly high. Figure 3c confirms this speculation from two aspects. First, in each of the 2D scatter plots in Figure 3c, the dots distribute along the diagonal line, suggesting that the intensity of the chlorophyll autofluorescence signal is positively correlated with the intensity of the intracellular granularity signal of *H. pluvialis* green cells. Second, compared with the *H. pluvialis* green cells cultivated under blue light and blue/red light 1/2, the cells cultivated under red light and white light shifted toward higher intensities of FL4 (chlorophyll autofluorescence) signals and SSC (intracellular granularity) signals (Figure 3c), which were consistent with the results of Figure 3a,b.

The SSC signal determining intracellular granularity is thought to be related to starch accumulation in cells [30]. It was thus assumed that starch contents in *H. pluvialis* cells grown under different light spectra were different. This assumption was proved by the starch content analysis results shown in Figure 3d. The red light group showed the highest cellular starch content, 2.6, 4.2 and 4.8 times higher than that of the white, blue/red 1/2 and blue light groups, respectively.

In sum, *H. pluvialis* green cells cultivated under red light and white light had a larger size, higher photosynthetic pigment content, higher starch content and higher intracellular granularity than those cultivated under blue light and blue/red light 1/2.

### 3.3. Effects of Light Spectra on the Chlorophyll a Fluorescence Parameters of H. pluvialis Green Cells

**Figure 4:**
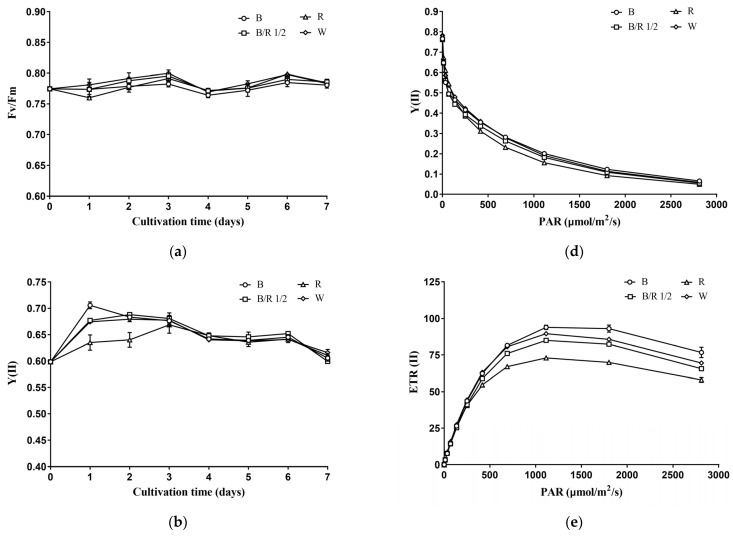

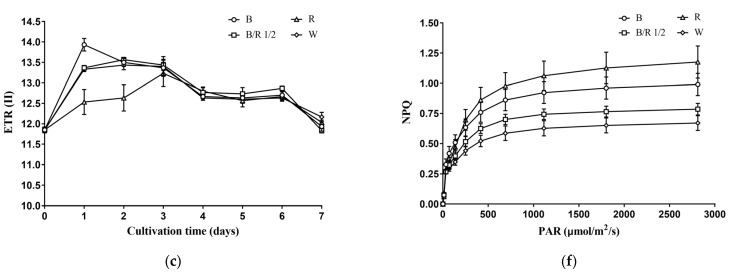
Effect of light spectra on the chlorophyll a fluorescence parameters of *H. pluvialis* green cells. (**a**) Trend of Fv/Fm with time. (**b**) Trend of Y(II) with time. (**c**) Trend of ETR(II) with time. (**d**) Trend of Y(II) with light intensity. (**e**) Trend of ETR(II) with light intensity. (**f**) Trend of NPQ with light intensity. In (**a**–**f**), B—Blue light; B/R 1/2—blue/red light 1/2; R—red light; W—white light. Results were presented as mean ± SD of the measurements of three biological replicates.

During the cultivation period, the Fv/Fm (maximum or intrinsic efficiency of PSII) of *H. pluvialis* green cells remained stable in a range of 0.76–0.80 (Figure 4a). Cultivation under different light spectra did not affect the Fv/Fm of *H. pluvialis* green cells (Figure 4a). The Y(II) (efficiency of PSII photochemistry) of *H. pluvialis* green cells varied between 0.60 and 0.70, and generally decreased with the extension of cultivation time (Figure 4b). In the early stage of cultivation, light spectra had significant effects on the Y(II) value of *H. pluvialis* green cells (Figure 4b). On the 1st day of cultivation, Y(II) was highest in blue light group, followed by blue/red light 1/2 group and white light group, as well as red light group (*p* < 0.05). On the 2nd day of cultivation, Y(II) was higher in blue light, blue/red light 1/2 and white light groups than in red light group (*p* < 0.05). In the middle and late stages of cultivation, the *H. pluvialis* green cells under different light spectra showed no significant difference in Y(II) value (*p* > 0.05). The trends of ETR(II) (linear electron transport rate) of *H. pluvialis* green cells with cultivation time and light spectra were consistent with those of Y(II) (Figure 4c).

At the end of cultivation, *H. pluvialis* green cells were collected and subjected to a series of light intensities to obtain the so-called light-response curves. As shown in Figure 4d, the Y(II) value of *H. pluvialis* green cells decreased with increasing light-intensity. At low light-intensity, *H. pluvialis* green cells cultivated under different light spectra showed no significant difference in Y(II). As the light intensity increased, the Y(II) values of *H. pluvialis* green cells cultivated under blue light, blue/red light 1/2 and white light became higher than those cultivated under red light. With further increase in light intensity, there was no significant difference in the Y(II) values of *H. pluvialis* green cells cultivated under different light spectra.

The ETR(II) of *H. pluvialis* green cells first increased with light intensity, and as the light saturation point was reached, ETR(II) gradually decreased with light intensity (Figure 4e). At low light intensity, there was no significant difference in the ETR(II) values among the *H. pluvialis* green cells cultivated under different light spectra. However, as the light intensity increased, the ETR(II) of *H. pluvialis* cells cultivated under blue light became higher than those cultivated under white light and blue/red light 1/2, and the latter values were higher than that cultivated under red light. Using the Eilers–Peeters (EP) model to fit the light response curves in Figure 4e, the light saturation points of *H. pluvialis* green cells cultivated under blue light, blue/red light 1/2, red light and white light were obtained: 454 ± 5, 426 ± 6, 313 ± 11, and 405 ± 1 μmol photons m^−2^ s^−1^, respectively.

As can be seen in Figure 4f, the NPQ (nonphotochemical quenching, an indicator of photoprotective ability) of *H. pluvialis* green cells increased with light intensity. In addition, at low light-intensity, there was no significant difference in NPQ of *H. pluvialis* green cells cultivated under different light spectra. At high light-intensity, the NPQ was the highest in the red light group, followed by the blue light group, the blue/red light 1/2 group and the white light group.

In summation, the photosynthetic performance of *H. pluvialis* green cells measured as chlorophyll a fluorescence was not significantly affected by light quality at the light intensity used for cultivation but was significantly affected by light quality at elevated light intensities.

### 3.4. Effects of Light Spectra on the Lipidome of H. pluvialis Green Cells

**Figure 5:**
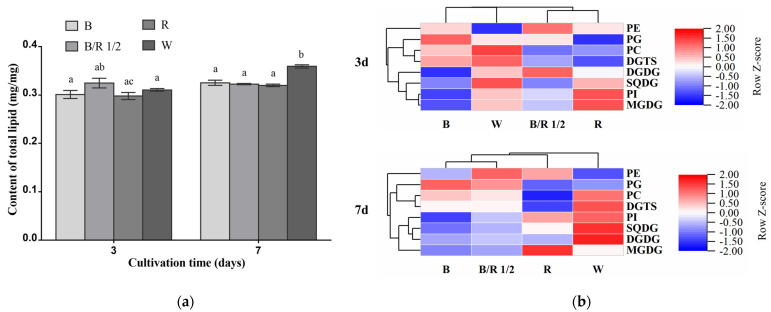
Effects of light spectra on the contents of total lipids and lipid classes in *H. pluvialis* green cells. (**a**) Total lipid content. The same lowercase letter means insignificant difference, while different lowercase letters mean significant difference. (**b**) Heat map of relative lipid class content under different light spectra. Row Z-Score scaling method was used for visualization in heat maps and allows for comparison of the content of the same single lipid class between different light spectra groups. Red color indicates comparatively high content, whereas blue color suggests comparatively low content. In (**a**,**b**), B—Blue light; B/R 1/2—blue/red light 1/2; R—red light; W—white light. Results were presented as mean ± SD of the measurements of two biological replicates.

The total lipid contents of *H. pluvialis* green cells grown under different light spectra were very close among each other in the exponential growth phase, accounting for about 30% of their dry cell weights (Figure 5a). As the cultivation extended to the stationary growth phase, the total lipid content of white light group became significantly higher than those of the other groups (Figure 5a, *p* < 0.05).

In the exponential growth phase, the blue/red light 1/2 group was characterized by higher contents of DGDG and PE compared with other groups. The red light group showed higher contents of MGDG and PI than other groups. The blue light group displayed higher content of PG than other groups. The content of SQDG, DGTS and PC, respectively, was the highest in the white light group among the four groups (Figure 5b). In the stationary phase, notably, the contents of many lipid classes including DGDG, SQDG, DGTS, PC and PI in the white light group became higher than those in the other light condition groups, respectively. The blue/red light 1/2 group was also characterized by higher content of PE than the other groups. The content of MGDG remained the highest in the red light group among the four groups. Under blue light, the content of PG in *H. pluvialis* green cells was still higher than that under other light conditions (Figure 5b). In this growth phase, the triacylglycerol TAG contents in *H. pluvialis* green cells grown under all light spectra were quite low and barely detectable (Figure S2). 

**Figure 6:**
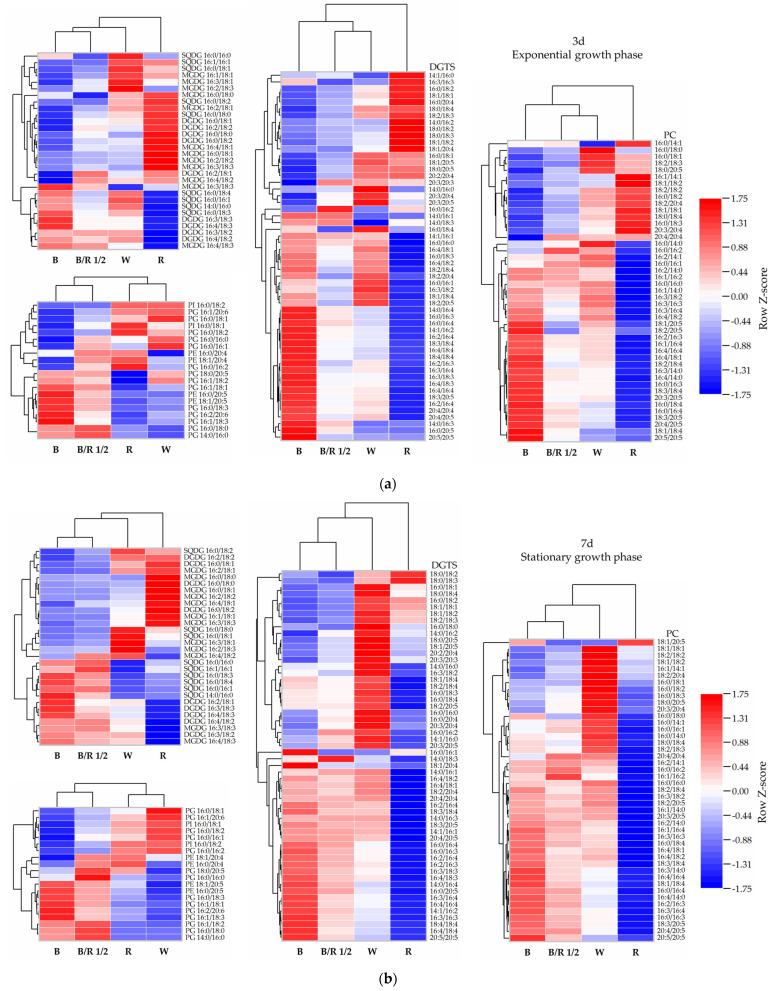
Heat map of relative contents of single lipid species in *H. pluvialis* green cells cultivated under different light spectra. (**a**) Exponential growth phase; (**b**) stationary growth phase. Row Z-Score scaling method was used for visualization in heat maps and allows for comparison of the content of the same single lipid species between different light spectra groups. Red color indicates comparatively high content, whereas blue color suggests comparatively low content. In (**a**,**b**), B—blue light; B/R 1/2—blue/red light 1/2; R—red light; W—white light.

In addition to total lipid content and lipid class content, the lipidome of *H. pluvialis* green cells was also found to vary with light spectra. Overall, it can be observed that the blue and red light caused opposite regulatory effects on the lipidome in *H. pluvialis* green cells at both the early exponential and stationary growth phases (Figure 6). The lipid profile of the fast-dividing cells grown under the blue/red light 1/2 was similar to that of the cells grown under the blue light, but dramatically different from that of the red light group.

At the exponential growth phase, compared with the slowly dividing *H. pluvialis* green cells in white light group (taken as control group), the fast dividing *H. pluvialis* green cells in blue/red light 1/2 group significantly upregulated the contents of 1 MGDG species (16:4/18:3), 1 DGDG species (16:2/18:2), 1 DGTS species (16:0/16:4), 2 PC species (18:1/18:4 and 16:0/18:4), 4 PG species (16:1/18:3, 16:0/18:3, 16:1/18:1 and 16:2/20:6) and 1 PE species (16:0/20:4) (*p* < 0.05). Meanwhile, the contents of a number of lipid species were significantly reduced in the blue/red light 1/2 group, including four MGDG species (16:2/18:3, 16:2/18:2, 16:2/18:1 and 16:1/18:1), 3 SQDG species (16:0/16:0, 16:0/18:2 and 16:0/18:1), eight DGTS species (18:1/20:5, 18:2/18:4,18:2/18:3, 18:1/18:4, 18:0/18:4, 16:3/18:2, 16:0/18:4 and 16:0/18:3), five PC species (18:2/20:4, 18:0/20:5, 18:2/18:3, 18:2/18:2, and 16:0/18:3), three PG species (16:0/18:2, 16:0/18:1, and 16:1/20:6) and one PI species (16:0/18:2) (*p* < 0.05). Among all the identified lipid species, DGDG 16:2/18:1, DGTS 20:3/20:3, DGTS 16:0/16:2, PC 20:4/20:4, PC 16:0/16:2, PG 16:0/18:0 and PG 14:0/16:0 presented the highest level, while MGDG 16:0/18:0, DGTS 16:3/16:3, DGTS 18:1/20:4, DGTS 16:0/18:4 presented the lowest level in the blue/red light 1/2 group compared to other light condition groups. However, the difference was not statistically significant. In general, the contents of single lipid species in the fast-dividing cells under blue/red 1/2 light remained at a moderate level compared with those in the slowly dividing cells under other three light spectra.

At the stationary phase, the contents of many lipid species, especially those of DGTS and PC, were enhanced in the white light group compared to the other groups, consistent with the trend revealed in Figure 5. Compared with the white light group, the blue/red light 1/2 group significantly downregulated the contents of four MGDG species (16:2/18:3, 16:2/18:2, 16:2/18:1 and 16:1/18:1), one DGDG species (16:2/18:2), two SQDG species (16:0/18:2 and 16:0/18:1), nine DGTS species (18:1/20:5, 18:0/20:5, 18:2/18:4, 18:2/18:3, 18:1/18:4, 18:0/18:4, 16:0/20:4, 16:0/18:4 and 16:0/18:3), six PC species (18:2/20:4, 18:0/20:5, 18:2/18:3, 18:2/18:2, 18:0/18:4 and 16:0/18:3) and one PI species (16:0/18:2), while significantly upregulating the contents of only one DGTS species (16:0/16:4), two PC species (18:1/18:4 and 16:0/16:4), two PG species (16:0/18:3 and 16:2/20:6) and one PE species (16:0/20:4) (*p* < 0.05). Among the identified lipid species, PC 16:0/16:2, PC 20:4/20:4, PG 14:0/16:0 and PG 16:0/18:0 still showed the highest level in the blue/red light 1/2 group among the four groups, the same as in the case of exponential growth phase. It is interesting to note that although no difference in cell number and dry cell weight was observed between the white and red light groups in the stationary growth phase (Figure 1), remarkable differences in lipidome were still found (Figure 6b). Compared with the white light group, the red light group displayed statistically significant difference in the contents of 8 MGDG species (16:2/18:3 showing the highest fold change), 2 DGDG species (16:3/18:3 showing the highest fold change), 20 DGTS species (16:4/18:3 showing the highest fold change) and 20 PC species (16:4/18:1 showing the highest fold change) (*p* < 0.05).

## 4. Discussion

Light serves as both source of energy and signals for microalgal cells. As the source of energy, it is captured by a light-harvesting complex composed of pigments and proteins in microalgal cells to drive photosynthesis. *H. pluvialis*, as a green microalga, contains chlorophyll a, chlorophyll b and carotenoids. Both blue (420–450 nm) and red (600–700 nm) lights can be absorbed by chlorophyll a with absorption peaks at 430 and 665 nm, and by chlorophyll b with absorption peaks at 453 nm and 642 nm [31]. Showing red, yellow or orange colors, carotenoids do not absorb wavelengths in those regions but instead in the violet/blue and blue/green regions [32]. Overall, the red and blue lights are basically the most important wavelengths for the photosynthesis of *H. pluvialis* and most other algae. As signals, light is detected by the photoreceptors also composed of pigments and proteins in microalga to regulate cell growth and metabolism through an extremely complex regulatory network. A blue light photoreceptor, phototropin (*phot*), has been identified in *H. pluvialis* [33]. Four putative blue light receptor cryptochromes *HpCPH1, HpCRY-DASH1*, *HpCPF1* and *HpPHR2* have been identified in *H. pluvialis* [34]. In *Chlamydomonas*, an animal-type cryptochrome has been found to be able to perceive red light [35]. Previous studies have proposed that cryptochromes can also perceive green light and an additional sensing system exists in cells to mediate effects specific to green wavelengths [36]. It was thus speculated that *H. pluvialis* green cells, with their photoreceptors, could detect and respond to different light signals.

A normal cell cycle includes cellular growth, DNA replication, nuclear division and cellular division. At the end of cellular growth phase, the cell attains a critical size and cell division can thus occur even without the supply of external energy. This time point is called the commitment point (CP) [24]. Research has found that blue light delays *Chlamydomonas reinhardtii* cell division and results in larger cell sizes compared with red light [37]. The mechanism is thought to be related to the delayed attainment of CP and larger critical size under blue light than under red light, and cyclin-CDK complex is thought to play a role [21]. Further research on *C. reinhardtii* suggests that the light-quality effect is not merely limited to cellular growth phase [38]. Additional research on *Chlorella vulgaris* has shown that transcription of *minD*, a gene encoding a cell-division inhibitor protein, is significantly suppressed under red light compared to blue or white light [20]. Despite all of these factors, it remains unclear how light spectra specifically regulates the cell cycle through regulating the cyclin-CD complex or the expression of cell division inhibitors and how and in what way photoreceptors play a role in these processes.

It is interesting to note the present study disagrees with the results of the above studies on how light wavelengths alter microalgal cell division. Our study showed that blue light facilitated cell division compared with pure red light or white light. This means that light wavelengths may differentially regulate cell division in *H. pluvialis* and other green microalgae such as *C. reinhardtii* and *C. vulgaris*. Furthermore, compared with blue light or red light alone, we found that the co-presence of blue light and red light enabled the fastest cell division. This possibly indicates the multiple sensing functions of one photoreceptor or the existence of multiple photoreceptors in *H. pluvialis*.

Our observation does agree with the phenomenon that fast cell division and large cell size cannot be obtained simultaneously, as reported in the previous study [20]. In the present study, the fast-dividing cells obtained under mixed blue/red light or blue light exhibited smaller size, lower dry weight per cell, lower photosynthetic pigment content per cell, lower starch content per cell and lower intracellular granularity than those slowly dividing cells cultivated under the red and white lights. One possible explanation for such a phenomenon is that cell division is an energy-demanding process and can consume the majority of the cell’s energy reservoir [39]. As the cell division slows down, cells are able to accumulate storage compounds, suggesting an inverse relationship between chemical energy storage and energy expenditure for the normal operation of cell cycle [40]. In the present study, the slowly dividing cells grown under red and white lights did accumulate higher contents of starch and total lipids than those fast-dividing cells grown under blue and blue/red 1/2 lights. Overall, these results suggested that the altered cell-division pattern of *H. pluvialis* vegetative cells is accompanied with the cell morphological and biochemical changes in response to different light wavelengths.

Accompanying cell-division pattern adjustment, the lipidome variations in *H. pluvialis* green cells grown under different light spectra were also detected. In addition to their well-known roles in membrane structure construction, signaling and energy storage, the functions of lipids in cell division have also been noticed. During cell division, lipids are involved in signaling, regulation of protein structure and function, as well as structural and mechanical support [41]. Our study found that the relatively fast-dividing but small cells in the blue light group and the slowly dividing but large cells in the red light group showed opposite regulation of their lipid profiles. Specifically, the lipid species that were upregulated in red light group were often downregulated in the blue light group, and vice versa. It was thus suggested that a correlation existed between cell lipid profile and cell division pattern. Furthermore, the fastest-dividing cells in the blue/red light 1/2 group displayed a similar lipid profile to those in the blue light group, but remarkably different from those in the red light group. Notably, the contents of single lipid species in blue/red 1/2 light group were often at the moderate levels among the four light spectra groups. Previous research has shown that the lipid profiles were finely tuned in the dividing cells [42]. The present study thus proposed that a systematics or holism perspective was especially needed to understand that *H. pluvialis* green cells did not excessively upregulate or downregulate the contents of only one or several lipid species, but instead carefully regulated the contents of almost all lipid species to appropriate levels in order to facilitate fast cell division.

In the early stationary growth phase, the white light group was characterized by higher total lipid content, as well as higher contents of DGDG, SQDG, DGTS, PC and PI, but almost the same photosynthetic performance compared to other light spectra groups. Previous research shows that photosynthetic activity does not necessarily change with photosynthetic membrane lipids [17], but the latter can affect plants’ ability to cope with high light intensities [43]. Indeed, remarkable differences in NPQ were observed between different light quality groups at elevated light-intensities, as revealed by the light-response curves. This suggests that the *H. pluvialis* green cells grown under different light spectra might show different photoprotective abilities in the red stage where high light is usually applied to induce astaxanthin accumulation [15]. Therefore, further research is needed to evaluate the astaxanthin accumulation performance of *H. pluvialis* green cells with different morphologies and inclusions as they enter the red stage. Only on such a basis can we conclude with more confidence on which light wavelength is optimal for *H. pluvialis* cultivation.

Interestingly, in addition to differences in total lipid and starch contents, remarkable differences in lipid profile were observed between the white and red light groups in the stationary growth phase, even though the culture growth (in terms of both cell number and dry cell weight) was similar between the two groups. It has been proposed that metabolic acclimation is a preparatory process with prolonged effects that will finally lead to higher-level (such as cellular-level) changes [17]. From another perspective, we tended to believe that molecular-level processes such as starch and lipid metabolism were far more sensitive than cellular-level processes such as cell growth in response to environmental variations.

## 5. Conclusions

Division and morphology of *H. pluvialis* green cells were significantly influenced by light spectra. The existence of blue light, alone or with red light, promoted cell division, while pure red light and white light allowed for larger cell sizes, higher cellular pigment, starch and lipid contents, and finally enhanced biomass production. Additionally, light spectra exerted profound impacts on the lipid profiles of *H. pluvialis* green cells. The contents of most lipid species in the blue/red 1/2 light group, which showed the fastest cell division, remained at a moderate level compared with those under other light spectra, indicating the fast-dividing cells were featured by a fine-tuned lipid profile. Although no obvious difference in culture growth was found between red and white light groups, starch content and lipidome variations were still observed between them, suggesting that molecular-level processes such as metabolism are far more sensitive to environmental stimuli than higher-level processes.

## Data Availability

All data presented in this study are available within this article or Supplementary Materials. There are no special databases associated with this manuscript.

## Supplementary Materials

The following supporting information can be downloaded at: https://www.mdpi.com/article/10.3390/cells11121956/s1, Figure S1: Blue, red, infrared and white light spectra used in this study; Figure S2: Thin layer chromatography (TLC) analysis results of total lipids extracted from *H. pluvialis* green cells grown for 7 days under different light spectra.
