# Supplementary material for: Regulation of Light Spectra on Cell Division of the Unicellular Green Alga Haematococcus pluvialis: Insights from Physiological and Lipidomic Analysis
Source: Cells. 2022 Jun 17;11(12):1956. doi: 10.3390/cells11121956 (PMC9221946; doi:10.3390/cells11121956)
Supplement: Supplementary file 1 [file cells-11-01956-s001.zip › cells-1731873-supplementary.pdf]

Supplementary Materials

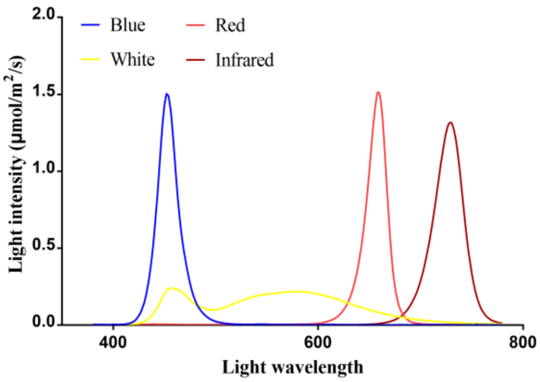

Figure S1. Blue, red, infrared and white light spectra used in this study (The blue and red light spectra presented here are mixed to obtain mixed blue/red lights).

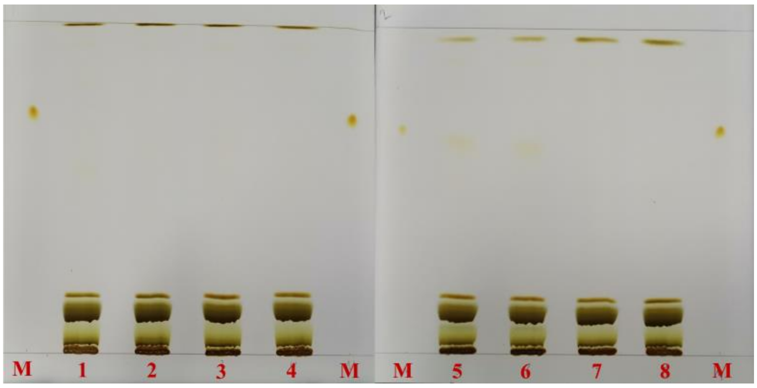

Figure S2. Thin layer chromatographic (TLC) analysis results of total lipids extracted from *H. pluvialis* green cells grown for 7 days under different light spectra. M: TAG standards used as marker; 1,2: Blue light group; 3,4: Blue/red 1/2 light group; 5,6: Red light group; 7,8: White light group. The spots scrapped off from TLC plates were analyzed by gas chromatography/mass spectrometry (GC/MS) after methyl esterification. Results showed that TAG accounted for 0.035±0.003%, 0.032±0.001%, 0.052±0.012% and 0.035±0.003% of the dry cell weights of *H. pluvialis* green cells grown under blue, blue/red 1/2, red and white lights, respectively.
